# Production of copolyesters of 3-hydroxybutyrate and medium-chain-length 3-hydroxyalkanoates by *E. coli* containing an optimized PHA synthase gene

**DOI:** 10.1186/1475-2859-11-130

**Published:** 2012-09-14

**Authors:** Xue Gao, Xiao-Xi Yuan, Zhen-Yu Shi, Ying-Ying Guo, Xiao-Wen Shen, Jin-Chun Chen, Qiong Wu, Guo-Qiang Chen

**Affiliations:** 1 MOE Key Lab of Bioinformatics, Department of Biological Science and Biotechnology, School of Life Science, Tsinghua-Peking Center for Life Sciences, Tsinghua University, Beijing, 100084, China; 2 Synthenome.com, Dingley Village, Melbourne, VIC, 3172, Australia; 3 Center for Nano and Micro Mechanics, Tsinghua University, Beijing, 100084, China

**Keywords:** PHB, Polyhydroxyalkanoates, PHA synthase, Codon optimization, Hairpin, Escherichia coli

## Abstract

**Background:**

Microbial polyhydroxyalkanoates (PHA) are biopolyesters consisting of diverse monomers. PHA synthase PhaC2_Ps_ cloned from *Pseudomonas stutzeri* 1317 is able to polymerize short-chain-length (scl) 3-hydroxybutyrate (3HB) monomers and medium-chain-length (mcl) 3-hydroxyalkanoates (3HA) with carbon chain lengths ranging from C6 to C12. However, the scl and mcl PHA production in *Escherichia coli* expressing PhaC2_Ps_ is limited with very low PHA yield.

**Results:**

To improve the production of PHA with a wide range of monomer compositions in *E. coli*, a series of optimization strategies were applied on the PHA synthase PhaC2_Ps_. Codon optimization of the gene and mRNA stabilization with a hairpin structure were conducted and the function of the optimized PHA synthase was tested in *E. coli*. The transcript was more stable after the hairpin structure was introduced, and western blot analysis showed that both codon optimization and hairpin introduction increased the protein expression level. Compared with the wild type PhaC2_Ps_, the optimized PhaC2_Ps_ increased poly-3-hydroxybutyrate (PHB) production by approximately 16-fold to 30% of the cell dry weight. When grown on dodecanoate, the recombinant *E. coli* harboring the optimized gene *phaC2*_*Ps*_*O* with a hairpin structure in the 5’ untranslated region was able to synthesize 4-fold more PHA consisting of 3HB and medium-chain-length 3HA compared to the recombinant harboring the wild type *phaC2*_*Ps*_.

**Conclusions:**

The levels of both PHB and scl-mcl PHA in *E. coli* were significantly increased by series of optimization strategies applied on PHA synthase PhaC2_Ps_. These results indicate that strategies including codon optimization and mRNA stabilization are useful for heterologous PHA synthase expression and therefore enhance PHA production.

## Background

Polyhydroxyalkanoates (PHA) are a family of biopolyesters accumulated by many bacteria, and have been studied to meet the various demands in chemical, material and special industries [1-6]. The structures and properties of PHA can be adjusted by controlling their monomer compositions [7,8]. On the basis of the carbon chain lengths of monomers, PHA are classified as short-chain-length PHA (scl PHA) and medium-chain-length PHA (mcl PHA) consisting of three to five and six or more carbon atoms in their monomers, respectively [7,9]. The differences in PHA monomers and compositions can strongly affect the properties and qualities of the polyesters [10-12]. Generally, PHA copolymers consisting of scl and mcl monomers are considered to have more potentials for applications due to their suitable properties including tensile strength, Young’s modulus, elongation at break and so on [13]. Particularly, the scl and mcl PHA copolymers consisting of high molar percentage of scl monomers (such as 3HB) and low molar percentage of mcl monomers have better application properties including high melting temperature and toughness as well as rapid crystallization process [14]. Many bacteria such as *Aeromonas hydrophila* and *Ralstonia eutropha* were reported to be able to produce scl and mcl PHA with different compositions and yields [15,16]. However, there are still difficulties to control the PHA monomer compositions when industrial fermentation processes are conducted for production of PHA [8].

One of the key factors that determine PHA monomers composition is the substrate specificity of the PHA synthase (PhaC). The production of scl and mcl PHA requires synthases with relatively wide substrate specificities, such as PhaC from *Aeromonas caviae*[17,18] and PhaC2_Ps_ from *Pseudomonas stutzeri* 1317 (named PhaC2_Ps_) [19]. These PHA synthases have been well characterized and expressed for production of scl and mcl copolymers [17,19]. For example, when expressed in a PHA synthase gene *phbC*_Re_ negative mutant *R. eutropha* PHB-4, PhaC2_Ps_ could confer on the host strain the ability to synthesize PHA with monomer compositions adjustable by altering carbon sources [19]. Furthermore, to achieve a high mole faction of 3-hydroxybutyrate (3HB) monomer in scl-mcl PHA copolymers for better thermal and mechanical properties, specific point mutagenesis was successfully applied [20], leading to mutated PHA synthase PhaC2_Ps_QKST that increased PHA accumulation up to 42 wt% consisting of 93 mol% 3HB in *R. eutropha* PHB-4. However, all of the PhaC2_Ps_ mutants have not yet been characterized in *E. coli* which is a well-developed cell factory for many fine products [21].

*E. coli* was successfully exploited for scl PHA [22] and scl-mcl PHA production [23,24]. Particularly, pathways were constructed to achieve scl-mcl PHA accumulation in *E. coli* using related or unrelated carbon sources [25-27]. However, the overall PHA yields were generally low probably due to inadequate supply of PHA precursors, and/or lower synthase activity during the polymerization process.

The stability of mRNA is one of the most significant factors that affect levels of protein synthesis, in this case, PHA synthase synthesis. It was reported that the secondary structures within 5’ untranslated regions (UTRs) of prokaryotic mRNA could act as mRNA stabilizers, prevent them from fast degradation by RNases and therefore promote translation [28]. Various rationally designed synthetic 5’ hairpin structures have been investigated and their half lives and effects in mRNA stability were measured [28,29], and it is significant to add different mRNA secondary structures in the 5’ UTR region for controlling the expression of recombinant genes. On the other hand, species-specific variations in codon usage are generally considered one of the major factors that affect heterologous protein expressions. If the concentrations of the *E. coli* tRNAs for the rare codons are insufficient to optimally translate mRNA [30], codon optimization could be effective to enhance protein expression [31,32].

To achieve high scl-mcl PHA production, the functions of the wild type *phaC2*_*Ps*_ and its recombinants in *E. coli* were evaluated in this study, and codon optimization and mRNA stabilization strategy were employed to enhance PHA synthase expression. The recombinant *E. coli* expressing the optimized PhaC2_Ps_ was proven to be able to produce scl-mcl PHA much more effectively than the wild type did.

## Results

### Analysis and optimization of the mutated PHA synthase PhaC2_Ps_QKST

**Figure 1:**
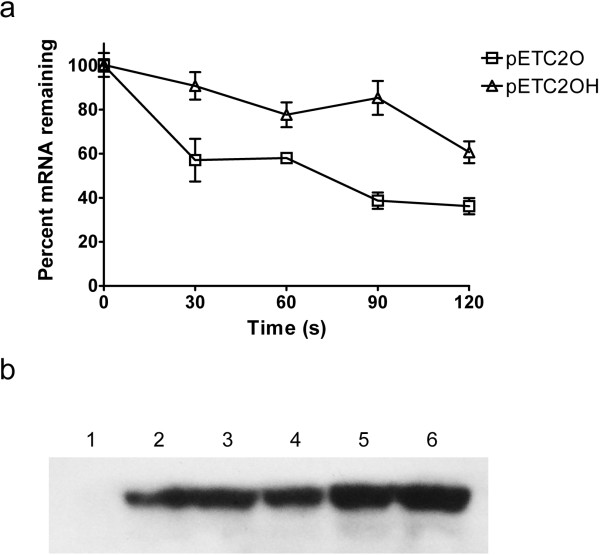
** Gene expression profiles and western blot analysis of PhaC2**_**Ps**_**s in recombinant*E. coli*harboring pET-based plasmids.** (**a**) The decay profiles of the transcripts of *phaC2*_*Ps*_*O* and *phaC2*_*Ps*_*OH* determined by quantitative RT-PCR. After adding rifampicin and incubating for 1 min, samples were iced at different timed intervals. The relevant abundances of mRNA compared to the *ompA* mRNA abundance were determined by qPCR, and the *y* axis showed the percentages of the remaining mRNA at timed intervals. Experiments were carried out in triplicate. (**b**) Western blot analysis of the PHA synthase PhaC2_Ps_ expressed in recombinant *E. coli* harboring pET-based plasmids. Lane 1 to 6: crude extracts from recombinant *E. coli* harboring pET28a, pETC2, pETC2QK, pETC2QKST, pETC2O, and pETC2OH, respectively.

**Figure 2:**
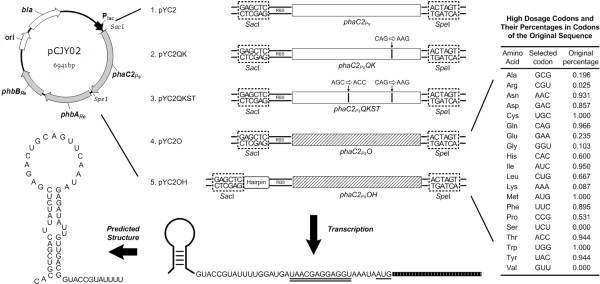
** Strategy for constructing DNA cassettes expressing different PhaC2**_**Ps**_**s for PHA production.** Five vectors containing different DNA cassettes of mutated *phaC2*_*Ps*_ under the control of a lac promoter were constructed. Genes *phaC2*_*Ps*_*QK and phaC2*_*Ps*_*QKST* are site-specific mutants of *phaC2*_*Ps*_ using the original codon strategy, and *phaC2*_*Ps*_*O* is the gene coding for PhaC2_Ps_QKST yet with its codon optimized (the selected codon for each amino acid and the percentage of this codon in the original coding sequence are listed on the right). In the fifth DNA cassette, DNA sequence coding for a hairpin structure [29] was inserted into the 5’ untranslated region of the gene. Double underlinines indicate the ribosome binding sites (RBS), and the single underlinine indicates translation start codon. Abbreviations: *bla:* beta-lactam resistance gene, *phbA*: β-ketothiolase gene, *phbB*: acetoacetyl-CoA reductase gene, *phaC2*_*Ps*_: a PHA synthase gene from *P. stutzeri* 1317.

In previous studies, the scl-mcl PHA production abilities of several mutants of PhaC2_Ps_ were studied [20]. A double mutant of PhaC2_Ps_ named PhaC2_Ps_QKST was shown to have higher scl-mcl PHA productivity and altered substrate specificity. When this mutated PHA synthase was expressed in *R. eutropha* PHB-4, the recombinant was reported to produce 42 wt% scl-mcl PHA [20]. However, the expression of this enzyme in *E. coli* was not high enough for producing large amount of PHA (Figure 1). Codon analysis of the original *phaC2*_*Ps*_*QKST* gene revealed that almost 60% codons were not preferred in *E. coli*. For example, in a total of 23 codons for lysine, 21 codons are AAG with a frequency of 27% in the *E. coli* genome, while only 2 codons are AAA with a frequency of 73%. Very rare codons such as AGG (Arg) and CUA (Leu) also exist. Thus, codon optimization of the *phaC2*_*Ps*_*QKST* gene may improve productivity by increasing protein expression. Therefore, PhaC2_Ps_QKST was optimized by “one amino acid-one codon” strategy, resulting in new PHA synthase gene *phaC2*_*Ps*_*O* (Figure 2). Also, the GC content of the coding sequence was adjusted from 66.7% to 58.3%.

To further enhance the expression level of the target protein, the hairpin structure pHP17 (Figure 2), which was reported to have the longest half life among a hairpin mRNA pool [29], was chosen to evaluate its effect on the expression of PhaC2_Ps_QKST. To do this, pHP17 was inserted to the upstream of the gene *phaC2*_*Ps*_*O*, resulting in *phaC2*_*Ps*_*OH* [GenBank: JX082171].

### Effect of the optimization strategies on mRNA degradation and protein expression of *phaC2*_*Ps*_

To assess the effect of codon optimization and hairpin stabilization, five pET28a-based plasmids were constructed expressing different *phaC2*_*Ps*_ genes, namely pETC2, pETC2QK, pETC2QKST, pETC2O, and pETC2OH. To demonstrate the effect of the hairpin introduction, the decay profiles of the optimized PHA synthase mRNAs with or without the hairpin structure were determined via quantitative real-time PCR. Figure 1a showed the percentages of the remaining mRNA at different time intervals. The mRNA with the hairpin structure (expressed in pETC2OH) appeared to be more stable than the one without it (expressed in pETC2O), indicating the hairpin structure had contributed to an enhanced level of gene expression.

The PHA synthase expression levels were further examined by western blot analysis (Figure 1b). While the original PhaC2_Ps_, the mutants PhaC2_Ps_QK and PhaC2_Ps_QKST were expressed with similar abundance, codon optimization of the gene significantly increased the protein expression level (lane 5). With hairpin structure inserted in front of the gene, the protein expression was further enhanced (lane 6).

### PHB accumulation in recombinant *E. coli* expressing optimized PhaC2_Ps_

To test the functions of different PhaC2_Ps_s for PHB production in *E. coli*, a series of plasmids were constructed harboring *phbA*, *phbB*, and different *phaC2*_*Ps*_ genes (Table 1). The five plasmids were named pYC2, pYC2QK, pYC2QKST, pYC2O, and pYC2OH, harboring PHA synthase genes including the original *phaC2*_*Ps*_, the two recombinants *phaC2*_*Ps*_*QK* and *phaC2*_*Ps*_*QKST*, the codon optimized *phaC2*_*Ps*_*O*, and *phaC2*_*Ps*_*O* with a hairpin coding region, respectively. The details are described in Methods. Other transcription and translation-related elements such as promoters and ribosome binding sites were maintained the same among those constructed plasmids to avoid differences resulting from those elements.

**Table 1:** Bacterial strains and plasmids

**Strains or plasmids**	**Relevant characteristics**	**Source or reference**
**Strains**		
*E. coli* JM109	*recA1, endA1, gyrA96, thi, hsdR17, supE44, relA1, Δ(lac proAB)/F’* [*traD36, proAB*^*+*^*, lac*^*q*^*lacZΔ*M15]	TaKaRa (Dalian, China)
*E. coli* LS5218	*atoC fadR*	[37]
**Plasmids**		
pBHR69	pBluscript SK- derivative, *phbA*_*Re*_ and *phbB*_*Re*_ cloned from *R. eutropha*	[38]
pCJY02	pBluscript SK- derivative, *phbA*_*Re*_ and *phbB*_*Re*_ cloned from *R. eutropha phaC2*_*Ps*_ cloned from *P. stutzeri 1317*	[39]
pSXWQK	pBBR1MCS-2 derivative, mutated *phaC2*_*Ps*_ Gln482→Lys (CAG→AAG)	[20]
pSXWQKST	pBBR1MCS-2 derivative, double mutated *phaC2*_*Ps*_ Ser326→Thr (AGC→ACC) Gln482→Lys (CAG→AAG)	[20]
pETC2	Wild type *phaC2*_*Ps*_ inserted into pET28a	This study
pETC2QK	Mutated *phaC2*_*Ps*_ inserted into pET28a	This study
pETC2QKST	Double mutated *phaC2*_*Ps*_ inserted into pET28a	This study
pETC2O	Codon optimized *phaC2*_*Ps*_*QKST* inserted into pET28a	This study
pETC2OH	Codon optimized *phaC2*_*Ps*_*QKST* with hairpin sequence inserted into pET28a	This study
pYC2	pBHR69 derivative, wild type *phaC2*_*Ps*_ of *P. stutzeri* 1317, plus *phbA*_*Re*_ and *phbB*_*Re*_	This study
pYC2QK	Mutated *phaC2*_*Ps*_ inserted into pBHR69	This study
pYC2QKST	Double mutated *phaC2*_*Ps*_ inserted into pBHR69	This study
pYC2O	Codon optimized *phaC2*_*Ps*_*QKST* inserted into pBHR69	This study
pYC2OH	Codon optimized *phaC2*_*Ps*_*QKST* with hairpin sequence inserted into pBHR69	This study

**Figure 3:**
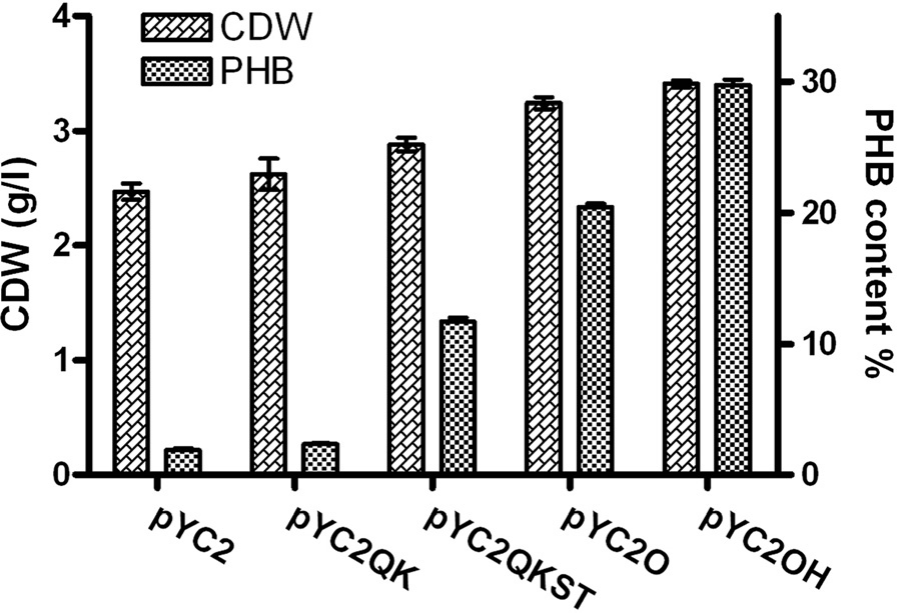
** PHB accumulation of*E. coli*JM109 expressing different PhaC2**_**Ps**_**s using gluconate as carbon source.** Data represent results for three independent experiments ± standard deviations. The cultivation was conducted in 500 ml shake flasks each having 50 ml MS medium containing 20 g/l gluconate and 100 mg/l thiamine. IPTG (0.1 mol/l) was used as an inducing agent for *lac* promoter and was added at 6 h after inoculation. After 48 h cultivation, strains harboring pYC2OH showed the highest CDW and PHB content (30 wt%, approximately 16-fold of the strains harboring pYC2), and the effect of codon optimization and hairpin stabilization was about 2.6-fold compared to the strain harboring pYC2QKST (p < 0.01).

The five constructed plasmids were transformed into *E. coli* JM109 to evaluate their functions of PHB accumulation, respectively. The shake flask study was performed using these recombinants grown on MS medium supplemented with thiamine. When 20 g/l gluconate was supplied as the sole carbon source, homopolymer PHB was synthesized under the catalysis of PhaC2_Ps_s after 48 h of cultivation (Figure 3). Shake flask experiment showed significant differences in PHB production and CDW among the recombinants (Figure 3). While native PhaC2_Ps_ only accumulated less than 2% PHB in cell dry weight (CDW) of 2.5 g/l, specific point mutagenesis of PhaC2_Ps_ increased PHB content to 2.4 wt% for PhaC2_Ps_QK and 12 wt% for double mutated PhaC2_Ps_QKST (Figure 3). Subsequently, the codon optimization of PhaC2_Ps_QKST showed a significant improvement in enzyme activity indicated by the enhanced PHB accumulation to 20.4 wt% CDW (Figure 3). The PhaC2_Ps_QKST with a hairpin structure that stabilized the mRNA further increased the PHB content to 30 wt%. The series of PhaC2_Ps_ optimization achieved approximately 15-fold improvement in PHB content in total, and the codon optimization and hairpin structure contributed approximately 2.6-fold. Along with the enhancement of PHA production, CDW was also increased from 2.5 g/l to 3.4 g/l in this process.

### Scl-mcl PHA production by recombinant *E. coli*

In order to study their abilities of scl-mcl PHA production, the recombinant plasmids pYC2, pYC2QK, pYC2QKST, pYC2O, and pYC2OH were transformed into *E. coli* LS5218, a FadR and AtoC (two regulators in fatty acid degradation) deficient strain [33]. The shake flask study was performed with these recombinant strains using MS medium supplied with 1 g/l yeast extract. When cultured with 8 g/l dodecanoate as a carbon source, the recombinant strains accumulated scl-mcl PHA with various concentrations and monomer compositions (Table 2). An obvious increase of PHA content from 0.81 wt% to 3.93 wt% (significance p < 0.01), along with a slight increase of CDW from 3.23 g/l to 3.59 g/l was observed. The recombinant harboring pYC2 could only accumulate 0.81 wt% PHA composed of 3HB, 3-hydroxyoctanoate (3HO) and 3-hydroxydecanoate (3HD), while the recombinant harboring the double mutated PhaC2_Ps_QKST accumulated PHA up to 1.79 wt% with an enhanced 3HB molar ratio at 84 mol%, and 3-hydroxydodecanoate (3HDD) was also detected. Furthermore, in the case of the codon optimized PhaC2_Ps_O, the recombinant strain accumulated 3.40 wt% PHA with monomers including 3HB, 3-hydroxyhexanoate (3HHx), 3HO, 3HD and 3HDD. Among those recombinants, the one harboring pYC2OH accumulated the highest PHA content of 3.93 wt%, in which the molar ratios of 3HB, 3HHx, 3HO, 3HD and 3HDD were 39.59, 5.80, 6.91, 45.46 and 2.24, respectively.

**Table 2:** **Scl and mcl PHA production by*E. coli*LS5218 harboring different PhaC2**_**Ps**_**s**^***a***^

**Plasmid**	**CDW**^***b***^**(g/l)**	**PHA content**^***c***^**(wt%)**	**Significantdifference**^***d***^	**PHA composition (mol %)**^***e***^				
				**3HB**	**3HHx**	**3HO**	**3HD**	**3HDD**
pYC2	3.23±0.03	0.81±0.06	-	32.35±1.16	ND ^*f*^	10.61±2.84	57.04±3.66	ND
pYC2QK	3.15±0.12	1.41±0.06	0.019	46.00±2.84	ND	6.90±0.53	47.10±2.82	ND
pYC2QKST	3.13±0.06	1.79±0.10	0.008	41.59±3.43	ND	6.28±0.56	49.93±2.77	2.21±0.09
pYC2O	3.39±0.04	3.40±0.05	0.004	39.49±0.36	5.93±0.05	6.97±0.06	45.33±0.24	2.27±0.02
pYC2OH	3.59±0.11	3.93±0.11	0.004	39.59±0.23	5.80±0.12	6.91±0.12	45.46±0.27	2.24±0.07

^*a*^ Cells were cultivated at 37°C and 200 rpm for 48 h as described in “Methods”. 8 g/l dodecanoate were added to the MS medium. Three parallel experiments were conducted for each datum.

^***b***^ CDW: cell dry weight.

^***c***^ PHA content: PHA contents are given as mass percentage of CDW.

^***d***^ Significant difference: p value calculated by student’s t-test, indicating the significant difference between the respective and the previous PHA contents.

^***e***^ 3HB, 3-hydroxybutyrate; 3HHx, 3-hydroxyhexanoate; 3HO, 3-hydroxyoctanoate; 3HD, 3-hydroxydecanoate; 3HDD, 3-hydroxydodecanoate.

^***f***^ ND: not detected.

To further investigate the effect of precursor supply in scl-mcl PHA production, different concentrations of gluconate were added as an additional carbon source to the recombinant *E. coli* LS5218 harboring plasmid pYC2OH. A better growth and higher PHA accumulation were observed when 5 g/l or 10 g/l gluconate was added compared to 20 g/l (Table 3), indicating a suitable balance between cell growth and PHA accumulation in this condition. 3HB fraction ranging from 52% to 96% in the polymers increased as more gluconate was provided (Table 3).

**Table 3:** **Scl and mcl PHA accumulation by recombinant*E. coli*LS5218 harboring pYC2OH grown in mixtures of gluconate and dodecanoate**^***a***^

**Concentration ofgluconate (g/l)**	**CDW (g/l)**	**PHA content (wt%)**	**PHA composition (mol %)**				
			**3HB**	**3HHx**	**3HO**	**3HD**	**3HDD**
5	3.73±0.02	4.18±0.16	52.39±3.40	5.16±0.54	20.24±1.11	14.89±1.75	7.32±0.70
10	3.47±0.33	3.99±0.35	70.11±3.12	4.50±0.31	14.16±0.72	7.01±0.62	4.22±0.08
20	2.10±0.10	3.36±0.18	96.33±0.78	ND	3.67±0.78	ND	ND

^*a*^ Cells were cultivated at 37°C and 200 rpm for 48 h as described in “Methods”. Mixed carbon sources consisting of different concentrations of gluconate and 8 g/l dodecanoate were added to the MS medium. Three parallel experiments were conducted for each datum.

## Discussion

With the development of synthetic biology, many functional modules are designed to be heterologously expressed in a host strain, and thus efficient and sometimes precise expression of a specific enzyme is generally required to meet the demand of coordination among multiple modules [34]. There are many possibilities to adjust expression levels of proteins in different situations, and codon optimization is generally considered a universal means to control or enhance heterologous protein expression [31,32]. Recent development in DNA synthesis and sequencing allows the rapid and accurate synthesis of large amount of DNA constructs at a lower cost, providing more accessibility to optimizing codon usage of heterologously expressed genes and to screening for suitable expression levels [35]. The “one amino acid-one codon” optimization strategy was employed in this study. PHB accumulation studies indicated that the mutagenesis and optimization of PHA synthase PhaC2_Ps_ cloned from *P. stutzeri* 1317 significantly enhanced the synthase polymerization activity, and thus promoted PHB synthesis (Figure 3). It was previously reported that PhaC2_Ps_ could achieve up to 40 wt% PHB accumulation when expressed in *R. eutropha* PHB-4 [19,20]. However, when expressed in *E. coli*, this PHA synthase could only accumulate trace amount of PHB (Figure 3). The series of site-specific mutagenesis of this synthase PhaC2_Ps_ was reported to achieve higher PHA accumulation and 3HB fraction in *R. eutropha* PHB-4 [20], and the same phenomenon could be observed when the mutated enzymes were expressed in *E. coli* (Figure 3). The double mutant PhaC2_Ps_QKST could enhance PHB accumulation approximately 6-fold compared to the wild type did, and this was partially due to the altered substrate-specificity [20]. Another 1.7-fold of PHB accumulation was observed in the case of codon-optimized synthase PhaC2_Ps_O (Figure 3). Since the specificity was not altered compared to PhaC2_Ps_QKST (the amino acids sequence was exactly the same), this result indicated that the increased PHB content was completely due to the enhancement of synthase expression.

The FadR-deficient strain *E. coli* LS5218 was used as the host to evaluate the scl-mcl PHA production driven by different PhaC2_Ps_s. When grown on MS medium supplemented with 8 g/l dodecanoate, all five recombinants harboring different plasmids could accumulate scl-mcl PHA (Table 2). Yet for strains harboring pYC2 or pYC2QK, only 3HB, 3HO and 3HD monomers were detected. Compared to the wild type PhaC2_Ps_, the two site-specific mutants PhaC2_Ps_QK and PhaC2_Ps_QKST increased PHA content and thus also the cell dry weight. Simultaneously, the substrate specificity was altered as a higher fraction of 3HB was observed, which was consistent with the previous report [20]. However, the overall PHA content was relatively low (1.79 wt%). After codon optimization of *phaC2*_*Ps*_*QKST*, the PHA content was significantly increased to 3.40 wt% and various PHA monomers were found including 3HB, 3HHx, 3HO, 3HD and 3HDD. Particularly, the 3HB monomer fraction was reduced slightly compared to the situation of PHA synthesis by PhaC2_Ps_QKST (Table 2), indicating that the monomer composition relies on multiple factors including the substrate specificity, the polymerization efficiency and the precursor supply. Both the PHB and scl-mcl PHA productions by the optimized PhaC2_Ps_QKST verified that the codon optimization strategy worked well in this condition (Figure 3 and Table 2). The replacement of rare codons such as AGG (Arg) and CUA (Leu) led to an significant increase in PHA production, indicating that the expression level of the key enzyme PHA synthase was strongly enhanced. It should be mentioned that there are other algorithms for codon optimization, and their functions on this specific case remain to be explored. It is expected that further optimization on PHA synthases can lead to wider substrate specificity and higher expression level, allowing the precise control of PHA polymerization.

Altering expression related factors such as promoters, ribosome binding sites, and mRNA stabilizers are possibilities to change the transcription and translation level of key PHA synthesis enzymes. In this study, a hairpin structure pHP17 which was reported to increase the half life of mRNA up to 19.8 min [29] was inserted in the 5’ UTR of *phaC2*_*Ps*_*O*, and it contributed another 1.5-fold in PHB accumulation (Figure 3), demonstrating that the degradation of the mRNA of *phaC2*_*Ps*_ is a significant factor that affects PHA accumulation. In the case of scl-mcl PHA production, the contribution of the hairpin structure was also significant (p < 0.01). However, since the main function of this structure is to increase mRNA stability, it is possible that the effect of a hairpin structure is largely dependent on the expression profiles in different conditions.

For the last version of the optimized PhaC2_Ps_ (codon optimized and hairpin inserted) the effect of different precursor supply situations to the monomer compositions of scl-mcl PHA was investigated. Compared to the presence of 20 g/l gluconate along with 8 g/l dodecanoate (Table 3), *E. coli* LS5218 harboring pYC2OH could accumulate slightly more PHA when 5 g/l gluconate or 10 g/l were added, and the CDW was also higher. This was probably due to a better balance that could be reached between cell growth and PHA accumulation under low gluconate concentration, which allowed proper coordination with the specificity and expression level of the PHA synthase. On the other hand, monomer compositions can be adjusted by providing different ratios of precursors coming from multiple metabolism pathways. Since *E. coli* has been well investigated and is relatively easy for genetic integration, we can expect efficient scl-mcl PHA production from unrelated carbon sources if precursor supplying pathways are introduced, and precise control of the carbon flux through these pathways would lead to PHA production with desired compositions. Furthermore, expression cassettes with high efficiency is often required for chromosomal expression since it is generally more difficult compared to plasmid system, therefore this investigation provides an optimized PHA synthase which may contribute to plasmid-free PHA production in *E. coli* in the future.

## Conclusions

In summary, a series of optimization strategies were applied on the PHA synthase PhaC2_Ps_ from *P. stutzeri* 1317, which is a PHA synthase with wide substrate specificities, to optimize its heterologous expression and PHA production in *E. coli*. The codon optimization and hairpin structure in the 5’-UTR of the mRNA were effective for optimizing PhaC2_Ps_ expression, and both the PHB and scl-mcl PHA production levels were significantly increased. The results indicated that these strategies provide a good opportunity for future PHA production with optimized yield and composition.

## Methods

### Optimization of the coding sequence and transcription region of target PhaC gene

For codon bias optimization, “one amino acid-one codon” strategy [36] was employed in this study to obtain an optimized translational region of the target gene. Codon preference in *E. coli* K-12 was selected and the codons of predicted highly expressed genes were chosen to replace rare ones. A hairpin structure which was reported to be able to increase mRNA stability [29] was introduced in the 5’ UTR of the mRNA for the target gene. The entire coding sequence of the optimized gene along with the hairpin region [GenBank: JX082171] was synthesized by Shanghai Qinglan Biotech Co., Ltd.

### Bacterial strains, plasmids, and general procedures for DNA manipulation

The main bacterial strains and plasmids used in this study are listed in Table 1. *E. coli* Trans1-T1 (Transgen) strain was used for plasmids construction mentioned in this study. *E. coli* JM109 and *E. coli* LS5218 [37] were used for evaluating the expression of the PHA synthase PhaC2_Ps_ and accumulating PHA with different compositions.

Plasmids related to PHA accumulation were a high-copy plasmid pBHR69 derivatives harboring *phbA* and *phbB* (originally cloned from *R. eutropha* H16) encoding β-ketothiolase and acetoacetyl-CoA reductase, respectively [38], together with different mutated *phaC2*_*Ps*_ genes. For plasmid constructions, genes were subcloned using standard PCR procedures, digested by respective restriction enzymes, and then ligated into the backbone of pBHR69. Specifically, DNA fragments coding for the optimized PhaC2_Ps_QKST with (termed *phaC2*_*Ps*_*OH*) or without (*phaC2*_*Ps*_*O*) a hairpin sequence were amplified using primer pairs YXXHF/YXXR or YXXF/YXXR (Table 4). Subsequently, the two fragments were digested by *Sac*I and *Spe*I, purified via gel electrophoresis, and finally inserted into pBHR69. The resulting plasmids were named pYC2OH and pYC2O, respectively. Similarly, genes encoding the wild type PhaC2_Ps_ and two site-specific mutants PhaC2_Ps_QK and PhaC2_Ps_QKST were subcloned from plasmids pCJY02 [39], pSXWQK and pSXWQKST [20] using primer pair 02SF and 02SR. These DNA fragments were digested by the same endonucleases and then ligated to pBHR69 backbone, leading to plasmids pYC2, pYC2QK and pYC2QKST, respectively. The constructs were verified by PCR using primer Tes02F and Tes02R followed by sequencing verification. To assess the effect of the optimization strategies on mRNA decay and gene expression, pET-based series plasmids were constructed: the five *phaC2*_*Ps*_ genes were cloned using primer pairs pET-F/R or pET-HF/R, digested by *Sac*I and *Hind*III, purified via gel electrophoresis, and finally inserted into pET28a. Restriction enzymes were purchased from Fermentas (China). Plasmids were sequenced by Biosune (Beijing, China).

**Table 4:** Oligonucleotide sequences

**Primers for construction and verification of plasmids**	
pET-F	TAAAGGGAACAAAAGCTGGAGCT
pET-R	GCCAAGCTTACGGATGTGAACGTAGGTAC??
pET-HF	GCCAAGCTTACGGATGTGAACGTAGGTAC
qPCR-OmpA-S	GTATGGCGTGCAGACACTAA
qPCR-OmpA-A	ACTGGTATTCCAGACGGGTAG
qPCR-phaC-S	TGATCCAGTACCGTCCGATGT
qPCR-phaC-A	GAAGACAGACCCCATTCACG
02SF	5’- ATTGAGCTCGTATTTTGGATGATAACGAGGAGGTAAATAATGCGAGACAAGCCCAATAG ^a^
02SR	5’- AATTACTAGTCCTCAGCGGATATGCACGTAGG -3’
YXXHF	5’- ATAATTGAGCTCCACGTCGACTTATCTCGAGACTG -3’
YXXF	5’- ATAATTGAGCTCGTATTTTGGATGATAACGAGGAG -3’
YXXR	5’- ATAATTACTAGTCCTTAACGGATGTGAACGTAGGT -3’
Tes02F	5’- ATTCTGTGGATAACCGTATTACC -3’
Tes02R	5’- GCACACCTTGTTGATGGTCATGG -3’

^a^Bases underlined indicate *Sac*I and *Spe*I restriction sites in the forward and reversed primers, respectively.

### Analysis of the mRNA decay profiles by quantitative real-time PCR

The recombinant cells (*E. coli* JM109 harboring pETC2O or pETC2OH) were cultured in LB medium. When OD600 reached 0.2, isopropyl β-D-1-thiogalactopyranoside (IPTG, 0.1 mol/l) were added and cells were cultured for another 2 h. Rifampicin was added to a final concentration of 50 μg/ml to inhibit RNA synthesis. After 1 min of incubation, culture samples were iced at timed intervals. Total RNA was isolated from each sample using the E.Z.N.A.® Bacterial RNA Kit (Omega Bio-tek). The purity and concentration of RNA were checked using a Nanodrop 2000 spectrophotometer (Thermo Fisher Scientific). Following cDNA synthesis was performed using the Quantscript RT Kit (Tiangen).

The mRNA decay profiles were determined by real-time PCR using the cDNA samples as the templates. The *ompA* gene was selected as the housekeeping gene. The real-time PCRs were performed using the SuperReal PreMix (SYBR Green) kit (Tiangen) with MxPro 3000P (Agilent Technology). The primers for real-time PCR are listed in Table 1, and the reaction condition was as follows: 95°C for 5 min; 95°C for 20 s, 55°C for 30 s, and 72°C for 30 s for 40 cycles.

### Western blot analysis

The recombinant strains were cultivated in LB medium with IPTG (0.1 mol/l) and then harvested by centrifugation. After resuspending the cells in PBS buffer, crude extracts were obtained by disrupting the cells with sonication. The concentrations of total proteins were determined with a BCA protein assay kit (Vigorous). Proteins (10 μg) in crude extracts were separated by sodium dodecyl sulfate-polyacrylamide gel electrophoresis (SDS-PAGE). Western blot analysis of PhaC2_Ps_ was carried out using anti His-tag mouse monoclonal antibodies (CW Biotech). Horseradish peroxidase (HRP)-conjugated anti-mouse antibodies (Santa Cruz Biotechnology) were used as a secondary antibody.

### Medium and culture conditions

Different strains of *E. coli* were grown in LB medium or on LB agar plates at 37°C. Antibiotics including ampicilin (100 mg/ml) and kanamycin (50 mg/ml) were added when necessary. Antibiotics were purchased from Sigma (Beijing, China). For PHA accumulation, shake flask experiments were carried out in a rotary shaker (NBS, Series 25D, New Brunswick, NJ, USA) at 200 rpm and 37°C using 500-ml conical flasks each containing 50 ml of medium. 20 g/l gluconate was used for the evaluation of optimized PhaC2_Ps_ for enhanced poly-3-hydroxybutyrate (PHB) synthesis, and 8 g/l dodecanoate plus 0 g/l, 5 g/l, 10 g/l, or 20 g/l gluconate was added as carbon sources in the case of scl-mcl PHA production. To achieve better utilization of dodecanoate, the flasks were shaken at 60 rpm, 45 °C immediately after sterilization in a rotary shaker until dodecanoate was scattered. Cells of *E. coli* JM109 or LS5218 were first cultured in LB medium for 12 h and then transferred to mineral salt (MS) medium containing different carbon sources at an inoculation volume of 4% for 48 h. The MS medium contained (per liter) 9.0 g Na_2_HPO_4_·12H_2_O, 1.5 g KH_2_PO_4_, 1.0 g (NH_4_)_2_SO_4_, 0.4 g MgSO_4_·7H_2_O and 2% (v/v) trace element solution as described in previous studies [40]. 100 mg/l thiamine was added into the MS medium when *E. coli* JM109 was used since this strain is thiamine-deficient. For scl-mcl PHA production, 1 g/l yeast extract was added for the culture of *E. coli* LS5218. IPTG (0.1 mol/l) was used as an inducing agent for lac promoter and was added at 6 h after inoculation.

### Gas chromatography (GC) analysis of PHA in dry cells

Cell dry weight (CDW) was evaluated by harvesting 30 ml cell cultures, centrifuged at 10,000 rpm for 10 min and then washed with distilled water when gluconate was added as sole carbon source. In the case of scl-mcl PHA production using dodecanoate as a carbon source, cells were washed twice with ethanol before washed with distilled water. CDW was measured after vacuum lyophilization (free of the remaining C12 carbon sources). The lyophilized cells and standard PHA samples were subjected to methanolysis in chloroform at 100°C for 4 h in the presence of 3% (v/v) H_2_SO_4_ to prepare samples for gas chromatography (GC) (SHIMADZU GC-2014C, Kyoto, Japan) equipped with 30-m HP-5 capillary column [41]. PHA contents were determined by analyzing the GC data.

### Statistical analysis

For statistical analysis, three parallel experiments were conducted and data collected were evaluated by mean ± SD. Student’s two-tailed t-test was performed to determine the significance of differences (**: p < 0.01, *: p < 0.05).

## Competing interests

The authors declare that they have no competing interests.

## Authors’ contributions

XG examined the mRNA stabilities and protein expression levels, performed the shake flask experiments and drafted the manuscript. XXY carried out the molecular manipulations and performed the preliminary shake flask experiments. ZYS designed the experiment and provide suggestions. YYG participated in plasmids construction. XWS carried out site-specific mutagenesis. JCC, QW and GQC supervised the study. All authors read and approved the final manuscript.
